# The Role of Engagement in a Tailored Web-Based Smoking Cessation Program: Randomized Controlled Trial

**DOI:** 10.2196/jmir.1002

**Published:** 2008-11-04

**Authors:** Victor J Strecher, Jennifer McClure, Gwen Alexander, Bibhas Chakraborty, Vijay Nair, Janine Konkel, Sarah Greene, Mick Couper, Carola Carlier, Cheryl Wiese, Roderick Little, Cynthia Pomerleau, Ovide Pomerleau

**Affiliations:** ^3^Henry Ford Health SystemDetroit, MichiganUSA; ^2^Group HealthSeattle, WashingtonUSA; ^1^University of MichiganAnn Arbor, MichiganUSA

**Keywords:** Internet, World Wide Web, smoking cessation, engagement

## Abstract

**Background:**

Web-based programs for health promotion, disease prevention, and disease management often experience high rates of attrition. There are 3 questions which are particularly relevant to this issue. First, does engagement with program content predict long-term outcomes? Second, which users are most likely to drop out or disengage from the program? Third, do particular intervention strategies enhance engagement?

**Objective:**

To determine: (1) whether engagement (defined by the number of Web sections opened) in a Web-based smoking cessation intervention predicts 6-month abstinence, (2) whether particular sociodemographic and psychographic groups are more likely to have lower engagement, and (3) whether particular components of a Web-based smoking cessation program influence engagement.

**Methods:**

A randomized trial of 1866 smokers was used to examine the efficacy of 5 different treatment components of a Web-based smoking cessation intervention. The components were: high- versus low-personalized message source, high- versus low-tailored outcome expectation, efficacy expectation, and success story messages. Moreover, the timing of exposure to these sections was manipulated, with participants randomized to either a single unified Web program with all sections available at once, or sequential exposure to each section over a 5-week period of time. Participants from 2 large health plans enrolled to receive the online behavioral smoking cessation program and a free course of nicotine replacement therapy (patch). The program included: an introduction section, a section focusing on outcome expectations, 2 sections focusing on efficacy expectations, and a section with a narrative success story (5 sections altogether, each with multiple screens). Most of the analyses were conducted with a stratification of the 2 exposure types. Measures included: sociodemographic and psychosocial characteristics, Web sections opened, perceived message relevance, and smoking cessation 6-months following quit date.

**Results:**

The total number of Web sections opened was related to subsequent smoking cessation. Participants who were younger, were male, or had less formal education were more likely to disengage from the Web-based cessation program, particularly when the program sections were delivered sequentially over time. More personalized source and high-depth tailored self-efficacy components were related to a greater number of Web sections opened. A path analysis model suggested that the impact of high-depth message tailoring on engagement in the sequentially delivered Web program was mediated by perceived message relevance.

**Conclusions:**

Results of this study suggest that one of the mechanisms underlying the impact of Web-based smoking cessation interventions is engagement with the program. The source of the message, the degree of message tailoring, and the timing of exposure appear to influence Web-based program engagement.

## Introduction

Web-based programming for smoking cessation is now reaching millions at a relatively low cost [1,2]. Moreover, some Web-based cessation programming has been tested in randomized trials and found to produce cessation rates that are similar to other far more expensive channels [3-6]. These early results are reflected by similar findings in eHealth programming for other health-related behaviors and disease conditions [1].

A consistently troubling finding, however, is the relatively low rate of long-term engagement produced by many Web-based programs [7]. Brief engagement with a Web-based program may not necessarily be an indication of failure. Participants may disengage from a program after successful behavior change. For example, in an effort to solidify a non-smoking identity, a successful quitter may disengage from a program so as not to be reminded of their previous smoking behavior. In a previous trial of a Web-based smoking cessation program [6], the number of cessation program Web pages opened was not a good predictor of 12-week cessation.

Eysenbach [7] discusses the need for developing a “science of attrition”, calling for studies examining the degree to which attrition is associated with program failure and the predictors of attrition. If program attrition is, indeed, related to failure, it makes sense to study participant characteristics that predict disengagement, and the impact of specific program components that encourage long-term engagement (ie, “stickiness”).

Operational definitions for “engagement” must also be defined. Danaher and colleagues [8] identify a number of ways in which exposure and engagement in Web-based health behavior change programs may be determined, including the number, duration, and pattern of visits to the site, and the number and types of pages viewed. The authors also point out that no single, universally accepted, measure exists.

This study, which uses a fractional factorial design with multiple treatment components [9,10], focuses on 3 questions: First, does engagement in Web-based smoking cessation program content influence long-term outcomes? Second, do user characteristics predict disengagement from the program? Third, do particular intervention strategies enhance engagement? These questions are addressed among smokers enrolled in a Web-based smoking cessation program within 2 large Health Maintenance Organizations (HMOs). The measure of engagement used in this study is the number of program sections opened.

## Methods

### Participants

Participants were recruited from the memberships of 2 Health Maintenance Organizations participating in the National Cancer Institute’s (NCI) Cancer Research Network (CRN): Group Health (GH) of Seattle, Washington, and the Henry Ford Health System’s Health Alliance Plan (HFHS) of Detroit, Michigan. Both GH and HFHS are not-for-profit health care delivery systems. Individuals were eligible to participate if: (1) they had smoked at least 100 cigarettes in their lifetime, currently smoked at least 10 cigarettes per day, and had smoked in the past 7 days; (2) were seriously considering quitting in the next 30 days; (3) were 21 to 70 years old; (4) were a member of GH or HFHS; (5) had home or work access to the Internet and an email account that they used at least twice weekly; (6) were not currently enrolled in another formal smoking cessation program or currently using pharmacotherapy for smoking cessation; and (7) had no medical contraindications for nicotine replacement therapy (NRT).

All participants in the study received access, free of charge, to an individually tailored smoking cessation program delivered via the Web, although specific intervention components received by participants varied by the experimental group to which they were assigned. All participants also received, free of charge, a 10-week supply of NRT patches. The purpose of the NRT provision was to minimize the potential confounding effects of adventitious differences in physiological addiction and to allow participants to focus on the cognitive-behavioral aspects of smoking cessation. A previous trial combining a Web-based behaviorally-tailored smoking cessation program with NRT demonstrated positive and relatively high rates of cessation 3 months post quit date [6]. The study protocol was reviewed and approved by the Institutional Review Board (IRB) of each collaborating institution and of the University of Michigan by January, 2004.

### Recruitment

Participants were recruited through a combination of individual- and population-level strategies between September, 2004 and July, 2005. Each of the 2 health care organizations identified likely current smokers via automated smoking status data collected during recent medical appointments, documentation of smoking in electronic medical charts, an internal list of smokers collected during prior research, or lists of patients with smoking-related conditions who had previously been prescribed cessation medications. Thus, all invitees were likely to have been recent smokers with a high probability of being current smokers. These likely smokers were prescreened using records of the health care organizations for minimal inclusion criteria (eg, age) and were sent a study invitation letter. Recruitment information informed the likely smokers that they would receive a free Web-based smoking cessation program and a free 10-week course of nicotine replacement therapy (NRT). Provision of NRT was predicated on eligibility and completion of the baseline assessment, but not continued participation in the program. Individuals who had not opted out of further contact or had not begun enrolling in the program at least 4 weeks after their initial invitation were sent a second ‘reminder’ mailing. Several population-level enrollment strategies were also utilized, including promotion of the study in the HMO newsletter and to HMO staff. Further description of participant recruitment procedures and the results of these procedures are presented in McClure et al [11].

### Data Collection, Randomization, and Follow-Up Procedures

Those invited to participate in the study were given a Web address (URL) and an identification code to enter a personalized website. After logging in, invitees were administered an eligibility survey, online consent, and baseline questionnaire. The intervention delivery system controlled the interaction with the participant by running a software script that collected data from the participant via an assessment and immediately produced appropriate (ie, tailored) cessation feedback based on those data. The baseline assessment assessed, and stored in a database, the participant’s smoking history, psychosocial, health, and demographic characteristics relevant to smoking cessation programming. A quit date within 3 weeks of the baseline assessment was also required. Immediately after the assessment, randomization was stratified by the HMO site automatically by the computer, invisible to the participant. Follow-up interviews administered 6 months post quit date were conducted using a computer-assisted telephone interview (CATI).

### Intervention Factors

The overall Web-based program and each experimental factor within the program were developed at the University of Michigan’s Center for Health Communications Research (UM-CHCR). The content of the program was based on cognitive-behavioral methods of smoking cessation and relapse prevention, including an appeal to motives for quitting, stimulus control, self-efficacy enhancement, and suggestions for coping with tempting situations and emotions. The intervention components selected for testing within this overall paradigm included outcome expectations, efficacy expectations, use of hypothetical success stories, personalization of the message source, and the timing of message exposure.

For 3 of these factors (outcome and efficacy expectations, success stories), the depth of tailoring was experimentally manipulated. By the term “tailoring” we refer to a process consisting of: (a) an assessment of individual characteristics relevant to smoking cessation, (b) algorithms that use the assessment data to generate intervention messages relevant to the specific needs of the user, and (c) a feedback protocol that delivers these messages to the smoker in a clear, vivid format. The Web-based program includes integrated cessation messages from multiple assessment responses to develop sentences and paragraphs written specifically for the user. For further description of the UM-CHCR’s tailoring process and examples of tailored feedback, the reader is referred to the UM-CHCR website [12].

Study participants received a variation of each of the 5 two-level intervention factors (1) depth of tailored outcome expectation feedback; (2) depth of efficacy expectations; (3) depth of success stories; (4) personalization of source; and (5) exposure schedule. Each of these factors is described in turn below.

#### Depth of Outcome Expectations

In this factor, the depth of tailored outcome expectation feedback was manipulated. Messages included statements tailored to personal and family health history, perceived health status, functional health status, monetary savings, and appearance, among other outcomes. Participants randomized to the high-depth tailored group received feedback and advice related to their specific motives for quitting. In addition, these participants received an overview of the balance between their intrinsic versus their extrinsic reasons for quitting. Participants in the low-depth tailored group received feedback related to their motives for quitting but did not make as many connections with existing health or lifestyle characteristics, nor was feedback regarding the balance between intrinsic and extrinsic motives provided.

#### Depth of Efficacy Expectations

Tailored efficacy messages addressed relevant barriers to quitting. Responses to high-risk situations, existing skills, and attributions for previous failures in quitting, along with smoking history and current smoking behavior were used to help build self-efficacy feedback. Those with previous cessation attempts, for example, were asked to consider these experiences in developing coping strategies for specific perceived cessation barriers. Participants randomized to the high-depth tailored group received feedback and advice focusing attention on their 2 most problematic individual barriers to quitting (for example, wanting to smoke when drinking coffee, when feeling stressed, or when spending time with friends and family who smoke). Highly tailored feedback also used information about the participant's home environment, family life, stress and coping levels, coping skills, and level of physical activity, among other unique characteristic traits to provide enhanced advice in dealing with the barriers addressed. Participants in the low-depth tailored group received less tailored content addressing 2 broader barrier topics cited by the smoker (for example, daily routines, negative emotion control, or social settings).

#### Depth of Success Stories

As part of the intervention, participants received a hypothetical story about an individual who successfully quit smoking. Low-depth success stories were tailored only to the participant’s name (ie, personalized) and gender. Participants randomized to high-depth success stories received a story that was tailored not only to their name and gender, but also to their age, ethnicity, marital status, smoking status of the spouse, number of cigarettes, biggest barrier to quitting, reason for wanting to quit, degree and type of social support, as well as whether the participant had children in the home, was physically active, and was working outside of the home.

#### Personalization of Source

In the introductory section welcoming a participant to the program, the highly personalized source condition included a photograph of, and supportive text from, the smoking cessation team of the HMO. It was written in a friendly manner, using words like "we" and "our team", and ended with a signature from the team. The low-personalized version included a photograph of a building representing the HMO institution, was written using words like "this organization", and did not include a closing signature.

#### Exposure Schedule

This manipulation compared the impact of providing the smoking cessation content in a single, large set of materials (equivalent to roughly 16 pages of text in a printed self-help guide) to that of breaking the materials into a series of weekly installations. Participants received the efficacy, outcome, success story, and source materials all at one time online or distributed over 5 weeks (efficacy messages were separated into 2 weeks) with email reminders to revisit the site when new content was made available. In both exposures, once content was available, it remained available throughout the study period.

### Experimental Design

This study was designed to identify the most active intervention components or “factors” from a large number of potentially relevant components [9,10]. A fractional factorial design with 16 arms allowed us to estimate all main effects and several pre-specified 2-factor interactions among the 5 intervention components. The study was intended primarily to test the impact of the 5 treatment components on 6-month smoking cessation outcomes. The results of this analysis are being presented in a separate paper (under review). However, the ongoing measurement of engagement in the program allows the determination of: (a) whether engagement with the program is associated with 6-month cessation, (b) characteristics of participants likely to disengage in the program, and (c) whether the treatment components tested in the study are related to engagement.

### Measures

#### Engagement

Engagement was determined through an automated assessment of the number of sections of the Web-based smoking cessation program opened. The sections of the program, described in the previous section, focused on particular treatment components, including outcome expectations, efficacy expectations, success stories, and message source. There were 2 efficacy expectation sections, creating a total of 5 sections that could have been opened by the participant. Program engagement was measured by the cumulative number of Web-based smoking cessation sections opened by the participant.

#### Tailoring Depth

To determine the impact of increasing tailoring depth on engagement, a score was created representing the number of high-depth tailored components received by the participant. Randomization of the 3 tailoring depth factors (outcome expectation, efficacy expectation, and success stories) allowed participants to receive a range of 0-3 high-depth tailored components.

#### Perceived Message Relevance

At the 6-month follow-up, a single-item measure, the degree to which the materials were found to be “written personally for me”, was asked. Messages tailored to specific needs and interests of the individual are often evaluated using this measure [13,14]. In recent research, Strecher, Shiffman, and West [15] found that the influence of Web-based tailored smoking cessation materials on subsequent abstinence was partially mediated by the participant’s perception that the messages were written for them.

#### Abstinence

The abstinence measure used in this study, collected 6 months following the participant’s self-identified quit date, is 7-day point prevalence abstinence (“Did you smoke a tobacco cigarette, even a puff, in the past 7 days?”). Abstinence was assessed by self-report during a telephone interview at 6 months post-quit date. Biochemical verification was not collected since it was considered impractical in this population-based study [16]. Moreover, there is general consensus that self-report is adequate in minimal-contact treatment studies when low demands exist to misrepresent one’s smoking status [17,18].

### Data Analysis

Logistic regression and analysis of variance (ANOVA) procedures were used to address the 3 questions of this study: (a) whether engagement with the program is associated with 6-month cessation, (b) characteristics of participants that predict engagement in the program, and (c) treatment components that predict engagement in the program.

The analysis examining engagement by 6-month cessation was conducted in 2 ways: a complete respondent (CR) analysis, and an intent-to-treat (ITT) analysis. The CR analysis focused on participants who answered the smoking cessation-related questions at 6-month follow-up. In the ITT analysis, all participants who were randomized to treatment, including those who failed to provide abstinence data for any reason, were included in the analysis. Non-respondents at follow-up in this case were considered treatment failures (ie, current smokers). The two remaining research questions were examined using baseline participant data and engagement data, which were collected from all baseline participants.

Exposure schedule, whether the programming was delivered over weekly installments or as a single grouping of sections, was considered a fundamental, structural feature of the Web-based programming. Therefore, in addition to examining this factor as a predictor of engagement, analyses were also stratified by this factor.

## Results

### Project Quit Recruitment and Follow-Up Response

During an 11-month recruitment period, 3256 people from both HMOs visited the website; 2651 (81% of website visitors) were screened for eligibility; 2011 (62% of website visitors) were eligible; and 1866 enrolled and were randomized to 1 of the 16 study arms (57% of website visitors). The primary reasons for ineligibility to the study were: did not smoke enough (26%), medical contraindications for NRT (23%), already enrolled in another smoking cessation program (16%), lack of adequate Internet/email access (14%), not currently enrolled in the HMO (10%), and currently using pharmacotherapy to quit smoking (8%).

Of these participants, 1415 (76%) responded to the 6-month follow-up computer-assisted telephone interview (CATI) and were included in the complete respondent (CR) analyses. A chi-square test was used to assess whether the non-response rate to the 6-month follow-up varied among the 16 treatment arms (cells of the fractional factorial design). No significant differences in non-response rates between intervention arms were found (*P* = .75).

### Participant Characteristics

Demographic, smoking, and psychosocial characteristics of enrolled participants by HMO are presented in Table 1. Possible differences in each of these baseline characteristics across the 5 experimental conditions were examined using analysis of variance (ANOVA). Of the 40 comparisons, significant differences at the *P* < .05 level (unadjusted for multiple comparisons) were found only for 2 baseline characteristics, motivation and self-efficacy, which were higher in the low- than in the high-tailored success story condition.

**Table 1:** Participant characteristics by HMO (blinded)

Participant characteristic	Site 1 (n = 986)	Site 2 (n = 880)	Total (n = 1866)
Age (mean years)	46.5	46.1	46.3
Gender (women)	59.4%	59.6%	59.5%
**Race**^a^			
African-American	3.2%	19.7%7.4%	11.0%
White	84.2%	72.9%	78.9%
Other	12.6%	7.4%	10.1%
**Education**			
≤ High school^b^	35.2%	37.3%	36.2%
> High school	64.8%	62.7%	63.8%
# cigarettes smoked/day (mean)^a^	21.1	22.7	21.8
Motivation (mean on 1-10 scale)	8.3	8.3	8.3
Self-efficacy (mean on 1-10 scale)	7.3	7.4	7.4

^a^ANOVA significant (*P* < .05) between HMOs

^b^This category includes vocational training

### Program Engagement and 6-Month Cessation

Using intent-to-treat criteria (treating 6-month non-respondents as smokers), the cumulative number of Web sections opened was related to subsequent smoking cessation (OR = 2.26; CI = 1.72-2.97) across the entire 0-5 range of sections opened. Each section opened, on average, contributed to an 18% higher likelihood of quitting smoking (OR = 1.18; CI = 1.11-1.24). Dichotomizing usage into “heavy” (3-5 sections opened) versus “light” (0-2 sections opened), a significant effect was also found: participants heavily engaged in the Web program had an average 6-month cessation rate of 37.4% while participants lightly engaged had an 27.3% cessation rate (X^2^ = 16.1; *P* < .001). Respondent-only analyses found similar, statistically significant effects. Including baseline levels of motivation and self-efficacy in the regression model did not influence the results.

### Participant Characteristics Predicting Program Engagement

Linear regression was used to analyze the relationship between participant characteristics and the number of sections opened (Table 2). Smokers who opened fewer sections tended to have less formal education, were younger, and were male. With the exception of HMO affiliation, these differences in engagement were found only in the weekly exposure condition.

**Table 2:** Program engagement^a^ of each intervention component by participant characteristics (n = 1866)

			Exposure Schedule			
			Single Exposure		Weekly Exposure	
Participant characteristic	# sections opened	F (*P* value)	# sections opened	F (*P* value)	# sections opened	F (*P* value)
**HMO**						
1	2.9	14.0 (*P* < .001)	3.2	6.5 (*P* = .01)	2.6	9.9 (*P* = .002)
2	2.2		2.9		2.2	
**Age**						
<40 yrs	2.5	11.4 (*P* < .001)	2.9	2.1 (*P* = .13)	2.0	14.4 (*P* < .001)
40-49 yrs	2.6		2.9		2.3	
>50 yrs	3.0		3.2		2.8	
**Gender**						
Female	2.8	5.9 (*P* = .02)	3.1	1.6 (*P* = .21)	2.5	7.2 (*P* = .008)
Male	2.6		3.0		2.2	
**Race**						
African-American	2.6	0.9 (*P* = .42)	2.9	0.3 (*P* = .71)	2.2	0.4 (*P* = .69)
White	1.7		3.1		2.4	
Other	1.8		3.1		2.5	
**Education**						
≤ High school	2.6	9.8 (*P* = .002)	2.9	2.5 (*P* = .11)	2.2	6.7 (*P* = .01)
> High school	2.9		3.2		2.5	
**# cigarettes/day**						
<20	2.8	0.6 (*P* = .56)	3.0	1.0 (*P* = .36)	2.5	2.1 (*P* = .12)
20	2.6		2.9		2.4	
>20	2.7		3.2		2.2	
**Motivation^b^**						
Low	2.8	2.3 (*P* = .13)	3.1	0.8 (*P* = .36)	2.5	2.9 (*P* = .09)
High	2.6		3.0		2.3	
**Self-efficacy^b^**						
Low	2.7	0.6 (*P* = .44)	3.2	2.3 (*P* = .13)	2.3	1.1 (*P* = .30)
High	2.7		2.9		2.4	

^a^Number of sections opened adjusted for baseline characteristics in the Table.

^b^Motivation and Self-efficacy measures were split at their means.

### Treatment Components Predicting Program Engagement

Table 3 presents the effects of each intervention component on program engagement. In this model, program engagement was regressed on each intervention component and the baseline variables of Table 1. More personalized source and high-depth tailored self-efficacy components were related to a greater number of Web sections opened. In addition, the single exposure that included all intervention components had the highest number of sections opened.

Stratifying by exposure schedule, 2 regression models were run, examining predictors of engagement with a Web program that included all intervention components simultaneously presented (“single”) versus a Web program that broke the materials into weekly installments (“multiple”). In the single condition, personalized source and highly tailored efficacy expectation messages were related to a higher number of sections opened. In the weekly exposure condition, no intervention components were related to the number of sections opened.

**Table 3:** Program engagement^a^ of each intervention component by intervention components (n=1866)

			Exposure Schedule			
			Single Exposure		Weekly Exposure	
Factor	# sections opened	F (*P* value)	# sections opened	F (*P* value)	# sections opened	F (*P* value)
**Source** High depth Low depth	2.9 2.6	10.2 (*P* = .002)	3.2 2.9	7.5 (*P* < .007)	2.6 2.5	2.4 (*P* = .12)
**Success story** High depth Low depth	2.7 2.7	0.0 (*P* = .97)	3.1 3.0	0.1 (*P* = .79)	2.5 2.6	0.1 (*P* = .76)
**Outcome expectations** High depth Low depth	2.7 2.8	3.2 (*P* = .07)	2.9 3.2	3.7 (*P* = .06)	2.5 2.6	0.3 (*P* = .60)
**Efficacy expectations** High depth Low depth	2.9 2.6	10.2 (*P* = .001)	3.2 2.9	6.6 (*P* = .01)	2.7 2.4	3.6 (*P* = .06)
**Exposure** Single Multiple	3.0 2.5	41.8 (*P* < .001)				

^a^Number of sections opened adjusted for baseline characteristics of Table 1.

**Figure 1:**
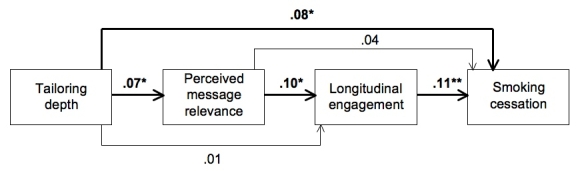
Path analysis of tailoring depth, perceived message relevance, longitudinal engagement, and smoking cessation. Numbers indicate standardized beta coefficients. Participants assigned to the weekly exposure condition (Complete Respondent analysis, n=725; *P < .05;**P < .01)

In a related study focused on smoking cessation outcomes [3], a significant relationship between tailoring depth, measured by the cumulative administration of high-depth success story, outcome expectation, and efficacy expectation components, and 6-month smoking cessation outcomes was found. Using this same tailoring depth measure, a path analysis model using linear regression was constructed for participants receiving the longitudinal exposure of intervention components. This path model includes tailoring depth, message relevance, engagement in the longitudinal program, and 6-month smoking cessation (Figure 1).

In this path model, the tailoring depth influenced perception of message relevance, which in turn, influenced longitudinal engagement in the sequentially delivered Web program. Engagement was related to smoking cessation. Tailoring depth also influenced smoking cessation outside of the hypothesized engagement pathway.

## Discussion

This research used a randomized trial to address 3 issues relevant to engagement in Web-based programming for health-related behavior change: (1) the degree to which engagement in program content influences 6-month smoking cessation outcomes; (2) characteristics of participants most likely to disengage with the program; and (3) intervention strategies that enhance engagement. These analyses found an average 18% increase in likelihood of quitting smoking for every Web section opened. The finding that engagement was associated with subsequent smoking cessation may not seem particularly surprising, though a null or even reverse result was possible if smokers who had successfully quit during the course of treatment decided to disengage from the program.

Identifying characteristics of participants more likely to disengage from the program offers targets for engagement efforts. Participants who were younger, were male, or had less formal education were more likely to disengage from the Web-based cessation program, particularly when the program sections were delivered sequentially over time. These sub-groups could, in the future, receive programming more specifically related to their needs and interests. In another recent study examining determinants of engagement in a Dutch Web-based weight management and lifestyle program, Verheijden and colleagues [19] found significantly lower engagement among younger users but not among less educated or male users. Together, these findings suggest that engagement patterns might vary by participant matter of the programming or perhaps by culture or other characteristics of the participants. The finding that older participants from both studies were more likely to remain engaged in the Web-based programming is interesting and relevant to programming targeted to seniors.

Particular components of the intervention influenced engagement with the Web-based programming. Both a more personalized source and highly tailored efficacy expectation messages were related to engagement when the Web program offered all content in a single large package. While message source is a classic focus in communications research, it is rarely examined in smoking cessation research. In this study, the source of the message was the participant’s health maintenance organization. While members of health maintenance organizations may perceive these organizations as untrustworthy due to a lack of openness and accountability [20], it is possible that a more personable message source may convey greater trustworthiness, leading to greater interest in the program. Further analyses showed that highly tailored messages related to self-efficacy and coping strategies for cessation may have promoted greater interim success or confidence, resulting in greater program engagement.

None of the individual intervention components influenced engagement when the sections of the program were distributed sequentially over a 5-week period. Since many Web-based programs are designed around a longitudinal engagement pattern, we wanted to focus further analysis on this issue, exploring the possibility that higher-depth tailoring might influence extended engagement. In a recent Web-based smoking cessation study, we found that message relevance partially mediated the influence of message tailoring on smoking cessation [6]. In other words, smokers receiving tailored versus untailored cessation materials were more likely to perceive the materials as personally relevant (ie, “written for me”), which in turn influenced greater cessation rates. In another recent study using functional magnetic resonance imaging (fMRI), we found that higher-depth tailored smoking cessation messages were associated with greater activation of a portion of the brain (medial prefrontal cortex) often associated with self-relevant activity [21].

In a path model constructed to explore this issue, perceived message relevance was associated with longitudinal program engagement. Message relevance, in turn, was influenced by greater depth of message tailoring. While other intervention strategies to influence longitudinal engagement exist (eg, email, IVR prompts), tailoring the message to specific needs and interests of the user appears to enhance perceived relevance, which in turn, appears to enhance engagement.

This study has a number of limitations. First, our measure of engagement was rudimentary. The number of Web sections opened does not describe the time, quality, or other aspects of engagement [8]. Second, our measure of personal relevance was based on a single questionnaire item and therefore participant to measurement error. Third, the sample of HMO members enrolling in a Web-based smoking cessation program is not generalizable to many other populations of smokers, including those unmotivated to quit and those who are uninsured.

In summary, this study found that: (1) engagement with a Web-based smoking cessation program was associated with subsequent cessation; (2) engagement was lower among younger, male, and less educated participants; and (3) engagement may be improved by including specific components to the intervention, particularly a more personalized source, and highly tailored messaging. Future research, with more detailed measures of engagement (eg, amount of time engaged with specific program components) and other engagement strategies (eg, email or IVR reminders to use the program) are likely to further our understanding of this issue. We believe that collecting multiple measures of engagement should be a routine part of all online interventions. A clear advantage of online interventions is the ability to measure engagement with relatively little effort, giving us greater insight into the process of program engagement and behavior change.

## Abbreviations

ANOVA: analysis of variance
CATI: computer-assisted telephone interview
CR: Complete Respondent
CRN: Cancer Research Network
fMRI: functional magnetic resonance imaging
GH: group health
HMO: health maintenance organization
HFHS: Henry Ford Health System
IRB: Institutional Review Board
ITT: intent-to-treat
IVR: interactive voice response
NCI: National Cancer Institute
NRT: nicotine replacement therapy
URL: uniform resource locator
UM-CHCR: University of Michigan’s Center for Health Communications Research
